# Expression Patterns and Functional Analysis of 11 E3 Ubiquitin Ligase Genes in Rice

**DOI:** 10.3389/fpls.2022.840360

**Published:** 2022-03-02

**Authors:** Huijuan Zhang, Dewei Zheng, Fengming Song, Ming Jiang

**Affiliations:** ^1^College of Life Science, Taizhou University, Taizhou, China; ^2^National Key Laboratory for Rice Biology, Institute of Biotechnology, Zhejiang University, Hangzhou, China

**Keywords:** rice, E3 ubiquitin ligases, biotic stress, abiotic stress, ROS, expression patterns

## Abstract

E3 ubiquitin ligases are involved in many processes, regulating the response to biotic and abiotic stresses. In this study, 11 E3 ubiquitin ligase genes from *Arabidopsis*, which were hypothesized to function in response to biotic or abiotic stresses were selected, and the homologous genes in rice were found. Their functions were analyzed in rice. These 11 E3 ubiquitin ligase genes showed different patterns of expression under different treatments. The BMV:OsPUB39-infiltrated seedlings showed decreased resistance to *Magnaporthe grisea* (*M. grisea*) when compared with BMV:00-infiltrated seedlings, whereas the BMV:OsPUB34- and BMV:OsPUB33-infiltrated seedlings showed increased resistance. The involvement of these genes in the resistance against *M. grisea* may be attributed to the regulation of the accumulation of reactive oxygen species (ROS) and expression levels of defense-related genes. Seedlings infiltrated by BMV:OsATL69 showed decreased tolerance to drought stress, whereas BMV:OsPUB33-infiltraed seedlings showed increased tolerance, possibly through the regulation of proline content, sugar content, and expression of drought-responsive genes. BMV:OsATL32-infiltrated seedlings showed decreased tolerance to cold stress by regulating malondialdehyde (MDA) content and the expression of cold-responsive genes.

## Introduction

Ubiquitination is a post-translational modification, involved in many processes. Ubiquitination is mediated by three sequential ubiquitin enzymes, E1 (the ubiquitin-activating enzyme), E2 (the ubiquitin-conjugating enzyme), and E3 (the ubiquitin ligase). The E3 ubiquitin ligase confers the specificity of the reaction and can either be single-subunit (including HECT, RING finger, and U-box domain family) or multi-subunit (such as SCF complex) (Morreale and Walden, 2016).

E3 ubiquitin ligases have been reported to function in several processes, including the responses to biotic and abiotic stresses (Ariani et al., 2017; Sun et al., 2019, 2022; Zhang et al., 2019; Du et al., 2021; Sharma et al., 2021). First, E3 ubiquitin ligases are involved in response to biotic stresses. The ATL subfamily that contains the conserved RING-H2 domain is activated by the elicitor and plays important roles in disease resistance, possibly through the regulation of the elicitor-signaling pathway, including chitin (Serrano and Guzman, 2004; Berrocal-Lobo et al., 2010; Ni et al., 2010; Chen et al., 2017; Deng et al., 2017). The U-box domain family of E3 ubiquitin ligases also plays important roles in defense response. The spl11 mutants show increased resistance to multiple fungal and bacterial pathogens (Yin et al., 2000; Zeng et al., 2004). AtPUB22, 23, and 24 act as negative regulators of biotic stresses (Cho et al., 2008; Trujillo et al., 2008). Moreover, E3 ubiquitin ligases are involved in the basic resistance of plants. Plants with overexpression of *HUB1* have a thickened cell wall to increase the resistance to *Botrytis cinerea* (*B. cinerea*), whereas the knockout mutants show a thinned cell wall to decrease the resistance to *B. cinerea* and *Alternaria brassicicola* (Dhawan et al., 2009). The overexpression of *OsBBI1* leads to increased accumulation of H_2_O_2_ and phenolic compounds, thicker cell wall, and increased resistance to *M. oryzae* (Li et al., 2011).

Secondly, E3 ubiquitin ligases are involved in response to abiotic stresses, including drought and cold stresses. E3 ubiquitin ligases function in response to drought stress, being either dependent on the ABA pathway (Zhang et al., 2005, 2015; Ko et al., 2006; Stone et al., 2006; Joo et al., 2016; Yang et al., 2016; Lim et al., 2017; Chapagain et al., 2018; Qin et al., 2020; Chen et al., 2021) or independent of it (Qin et al., 2008; Prasad et al., 2010; Suh and Kim, 2015; Wu et al., 2015). Moreover, E3 ubiquitin ligases also function in response to cold stress. In *Arabidopsis*, HOS1, AtATL78, and AtATL80 negatively regulated the tolerance to cold stress (Lee et al., 2001; Dong et al., 2006; Kim and Kim, 2013; Suh and Kim, 2015), whereas PUB25 and PUB26 positively regulated the tolerance to cold stress (Wang et al., 2019). OsDIRP1 positively regulated the tolerance to cold stress in rice (Cui et al., 2018) while OsATL38 negatively regulates the tolerance to cold in rice (Cui et al., 2022).

E3 ubiquitin ligases comprise a large protein family and are encoded by several genes (Bhaskar and Joemar, 2020); for example, more than 1,200 genes in *Arabidopsis* encode E3 ubiquitin ligases. This large number indicates the importance of E3 ubiquitin ligase. In this study, the functional analysis of 11 E3 ubiquitin ligase genes in rice was performed. The expression levels of some ubiquitin ligase genes were induced by one or several tested treatments, using different models. The silencing of the *OsPUB34* and *OsPUB33* led to increased resistance to *M. oryzae*, whereas the silencing *OsPUB39* led to increased resistance. The silencing of *OsPUB33* led to increased tolerance to drought stress, whereas that of *OsATL69* led to decreased tolerance, possibly through regulation of the proline content, sugar content, and expression levels of drought-responsive genes. Finally, the silencing of *OsATL32* led to decreased tolerance to cold stress, possibly through regulation of the MDA content and expression levels of cold-responsive genes.

## Materials and Methods

### Conditions for Plant Growth and Treatments Used

The rice cultivar Yuanfengzao, a pair of isogenic lines (H8R and H8S), and IR64 were used in this research for various purposes. The cultivar Yuanfengzao was used for the analysis of gene expression in response to hormone treatments and abiotic stress. H8R and H8S were used for the analysis of gene expression following inoculation with *M. grisea*. IR64 was used for VIGS infiltration. In the hormone treatment, 2-weeks-old Yuanfengzao seedlings were treated with 1.5 mM salicylic acid (SA, pH 6.5), 100 μM jasmonic acid (JA), 100 μM 1-amino cyclopropane-1-carboxylic acid (ACC), and 100 μM abscisic acid (ABA) (Sigma-Aldrich, St. Louis, United States). The leaves of the control group were sprayed with the same volume of water or 0.1% ethanol.

*M. grisea* (strain 85-14B1, race ZB1) was cultivated on oatmeal medium at 25°C for 10 days. The spores were collected and resuspended in water to a final concentration of 5 × 10^5^ conidia/mL with 0.02% Tween-20. Then, the spore solution was sprayed on the leaves of H8R and H8S (Luo et al., 2005). Leaf samples were collected at the indicated time points and stored at −80°C until further use.

For extreme temperature stress, 3-week-old plants were subjected to temperatures of 42 and 4°C. For the drought stress, hydroponic 3-week-old plants were placed on the floor of the frame in the greenhouse for growth after water on their root surface was absorbed by filter paper. For salt stress, hydroponic 3-week-old plants were transferred to 200 mM NaCl solution. Then, the samples were collected at the indicated time points (Hong et al., 2016). All the above-mentioned seedlings were grown in a room at 28°C, with a photoperiod of 14 h light/10 h dark. IR64 was used for VIGS assays, and the infiltrated seedlings were placed in a room at 24°C, with a photoperiod of 14 h light/10 h dark.

### Vector Construction and VIGS

The 200–400 bp fragments of target genes were constructed into BMV vector and confirmed by sequencing. The obtained recombinant plasmids were transformed into *Agrobacterium tumefaciens* strain C58C1 by electroporation using a GENE PULSER II Electroporation System. The agrobacteria confirmed by colony PCR were cultivated in a liquid YEP medium containing corresponding antibiotics at 28°C overnight. The bacteria were collected and resuspended in induction buffer (10 mM MgCl_2_, 10 mM MES, 200 μM acetosyringone, pH 5.7) and kept at 28°C for 5 h. The induction was stopped by centrifugation. The cells were then resuspended in an infiltration solution (10 mM MES, 10 mM MgCl_2_, 0.4 g/L L-cysteine, 0.15 g/L DTT, 0.75 mg/L silver nitrate, and 15 μL Silwet-77 in 10% YEP) and incubated at 28°C until the OD_600_ value reached 2.0. These treated cells were then mixed with the same volume of agrobacteria harboring pC13/F1 + 2 before vacuum infiltration. About 8–10-day-old IR64 seedlings were submerged completely in the mixed *Agrobacterium* suspension with vacuum infiltration for 7 min under a pressure of 20 kPa (model no. Rocker 410, Xiamen B&C Instrument Co., Ltd., China). Then, these plants were placed in a room at 24°C, with a photoperiod of 14 h light/10 h dark, and were recorded as BMV:target gene-infiltrated seedlings. The BMV:empty vector was transformed to seedlings as control, which were recorded as BMV:00-infiltrated seedlings (Zhang et al., 2021).

### qRT-PCR

RNA was extracted using the Trizol reagent (Invitrogen, Shanghai, China) according to the manufacturer’s instructions. cDNA was obtained using AMV reverse transcriptase (TaKaRa, Dalian, China) according to the manufacturer’s instructions. The qRT-PCR was performed using SYBR Premix Ex Taq™ (TaKaRa, Dalian, China) in a CFX96 real-time PCR detection system (BioRad, Hercules, CA, United States) according to the manufacturer’s instructions. The sequence of primers used in this study can be found in Table 1.

**TABLE 1:** The list of primer sequences used in this study.

Primers	Sequences (5′–3′)
*OsPUB39*-qRT-F	CCAGAGATATCGTTGCTGAGAC
*OsPUB39*-qRT-R	GATCGGGCACACGAAGTT
*OsATL17*-qRT-F	AAGCGGCGATCATCAACTAC
*OsATL17*-qRT-R	CGACCGCACAACCACAA
*OsPUB34*-qRT-F	CGAGGGAGATGCTCAAGATG
*OsPUB34*-qRT-R	GAGGGTAGGAAGCATTCAAGT
*OsPUB33*-qRT-F	CCTCGGCAAGGACAATGG
*OsPUB33*-qRT-R	TTCAGGAGGAGGATGGCATA
*OsPUB46*-qRT-F	CGATGCCTCTGTCACTTCTT
*OsPUB46*-qRT-R	GCGTTCTTCTCGAACTCGT
*OsRNF4*-qRT-F	GGCAGTAGTTGATCTGGAAGTAG
*OsRNF4*-qRT-R	TCTGGAGAGAGGCAGTGTATAA
*OsATL101*-qRT-F	CTACTACGCGACCAACTTCAG
*OsATL101*-qRT-R	GAAGAAGCCGAGGAAGAAGAA
*OsATL32*-qRT-F	CGAACAAGGGCGTCAAGA
*OsATL32*-qRT-R	GAACTCCACGAGGCAGATG
*OsATL9*-qRT-F	GCATCTTCCGCAATGTGTTC
*OsATL9*-qRT-R	TCAGACGCATCGTTCAACTC
*OsATL69*-qRT-F	CCGTTGGTGGTGAGCAA
*OsATL69*-qRT-R	TCTCTTGGCGTAGAGGTAGAG
*OsPUB31*-qRT-F	GGGTGAAGACCAAGGAGAAG
*OsPUB31*-qRT-R	TGGGTAAAGCGCCAAGAA
*OsPUB39*-vigs-F	ATACCTAGG GCGCTCACGGTGTTCTTCCC
*OsPUB39*-vigs-R	TATCCATGG TCGCTCGTCCCCTTGTCGG
*OsATL17*-vigs-F	TATCCATGG GACGACGACGACCACCACCA
*OsATL17*-vigs-R	ATACCTAGGCCACCACCTTCCCTTGACAGC
*OsPUB34*-vigs-F	ATACCTAGG GCGGGAGGAGCTGATGGCT
*OsPUB34*-vigs-R	TATCCATGG TTCCCTGCATCCGCGAGA
*OsPUB33*-vigs-F	ATACCTAGG GCTCATCCAGGCGTGGTGC
*OsPUB33*-vigs-R	TATCCATGGCGGACGGCTTGAGGGAGTAGA
*OsPUB46*-vigs-F	ATACCTAGG CTCCGCAGCCTCATCTCCCA
*OsPUB46*-vigs-R	TATCCATGG CGCCTCTTGTTCGCGTCCTC
*OsRNF4*-vigs-F	ATACCTAGG GCCTGTGGCAGTAGTTGA
*OsRNF4*-vigs-R	TATCCATGG AAGGTAAATACGGTGGAAAT
*OsATL101*-vigs-F	ATACCTAGGCCTCATGCTTCTCCTCCTGCTC
*OsATL101-vigs-R*	TATCCATGG CGCCCTTGACGGACTTGTGC
*OsATL32*-vigs-F	ATACCTAGG AACAAGGGCGTCAAGAAGGA
*OsATL32*-vigs-R	TATCCATGG ACGAGCACGCGGCGGCACGA
*OsATL9*-vigs-F	ATACCTAGGGCAGCCACATCTACCACCAGG
*OsATL9*-vigs-R	TATCCATGGGCGACCAAAGCACGAGAACAC
*OsATL69*-vigs-F	ATACCTAGG AGGAGGCGCTCGAGTGCGCG
*OsATL69*-vigs-R	TATCCATGG TTGGCGACGTCGTCGTGGGC
*OsPUB31*-vigs-F	ATACCTAGG TGCCGTCCTACTTCGTCTGCC
*OsPUB31*-vigs-R	TATCCATGG TGCCCTGCTATCCTCGCACTCC
*OsActin*-qRT-F	A AGCTGCGGGTATCCATGAGA
*OsActin*-qRT-R	GCAATGCCAGGGAACATAGTG
eEF1-qRT-F	CAACCCTGACAAGATTCCCT
eEF1-qRT-R	AGTCAAGGTTGGTGGACCTC
28s rDNA-qRT-F	TACGAGAGGAACCGCTCATTCAGATAATTA
28s rDNA-qRT-R	TCAGCAGATCGTAACGATAAAGCTACTC
*OsLOX1*-qRT-F	AAACGCTCGCTGGCATCAAC
*OsLOX1*-qRT-R	ATCGCCTCCTCCACCGTCAT
*OsNH1*-qRT-F	GCGGCGTCTCCTTGATGTCCTT
*OsNH1*-qRT-R	CGAGTTGTGGGTCCCTTCTTTC
*OsPR1a*-qRT-F	TCGTATGCTATGCTACGTGTTT
*OsPR1a*-qRT-R	CACTAAGCAAATACGGCTGACA
*OsPR3*-qRT-F	CACATACTGCGAGCCCAA
*OsPR3*-qRT-R	TTGTAGGTGATCTGGATGGG
*OsWRKY45*-qRT-F	CGGGCAGAAGGAGATCCAAAACT
*OsWRKY45*-qRT-R	GCCGATGTAGGTGACCCTGTAGC
*OsAP37*-qRT-F	AAGTGACTCCGACTCCTCGTC
*OsAP37*-qRT-R	GTTCAGATCCAGATCGAAAGCT
*OsbZIP23*-qRT-F	GGAGCAGCAAAAGAATGAGG
*OsbZIP23*-qRT-F	GGTCTTCAGCTTCACCATCC
*OsPP2C68*-qRT-F	CGCAGCTCCGACAACATCT
*OsPP2C68*-qRT-R	GCTGGGTGACACTCTCTCTACAAG
*OsRAB21*-qRT-F	CCACGGCACCGGGATGACC
*OsRAB21*-qRT-R	AGCTTCTCCTTGATCTTGTCCA
*OsERD1*-qRT-F	ACTGTAGTATTACTTGATGAGATA
*OsERD1*-qRT-R	CAATATTTGATGTCATGACAAT
*Myb*-qRT-F	ACGGCGGTGGGATTTCTTA
*Myb*-qRT-R	GCGATGCGAGACCACCTGTT
*CDPK7*-qRT-F	AACATGCCCGATGCTTTTCTT
*CDPK*-qRT-R	ATTGTTCTTCGTCCGACTCCC
*Fer1*-qRT-F	GGGAAAGGGAAGGAGGTGCT
*Fer1*-qRT-R	GTAGGCGAAAAGGGAGTGGT
*Trx23*-qRT-F	GTTCCCTGGTGCTGTCTTCC
*Trx23*-qRT-R	GCTTCACGATGGTGTTCTGG
*Lti6a*-qRT-F	CGGCGTCTTCTTCAAGTTCG
*Lti6a*-qRT-R	TGAGCAGCAAGCAGATCCAG

### Disease Assay of *M. oryzae*

About 5 μL of the *M. oryzae* spore solution was placed on the surface of leaves of 4-week-old silenced plants, which had been placed on a wet cheesecloth. Then, the inoculated leaves were kept in high humidity in a room for the growth of the silenced seedlings. After 7 days, photographs were taken, and the lesion sizes were recorded. For the analysis of the expression of defense-related genes and the quantification of *M. oryzae* in plants, we used the whole plant assay. It was carried out using the same method as done on the H8R and H8S seedlings.

### Abiotic Stress Tolerance Assay

For the analysis of drought stress, 4-week-old seedlings of BMV:OsATL69-infiltrated plants and BMV:00-infiltrated plants in the same pot were withheld from water for 10 days before re-watering, whereas the BMV:OsPUB33-infiltrated plants were withheld for 15 days. After 12 days, the survival rate, water loss, proline content, and sugar content were measured (Bates et al., 1973; Hong et al., 2016). For the analysis of cold stress, 4-week-old seedlings of BMV:target gene-infiltrated plants and BMV:00-infiltrated plants in the sameed that their expression decreased ied that their expression decreased i pot were kept at 4°C for 2 days before recovering to normal growth condition (Huang et al., 2016). The survival rate, MDA content, electrolyte leakage, chlorophyll content, and the expression levels of cold-responsive genes were analyzed as reported before (Hong et al., 2016).

## Results

### The Expression Levels of *OsPUB39*, *OsPUB34*, and *OsPUB33* Were Strongly Induced by the Inoculation of *M. girsea* and Hormone Molecules

**FIGURE 1:**
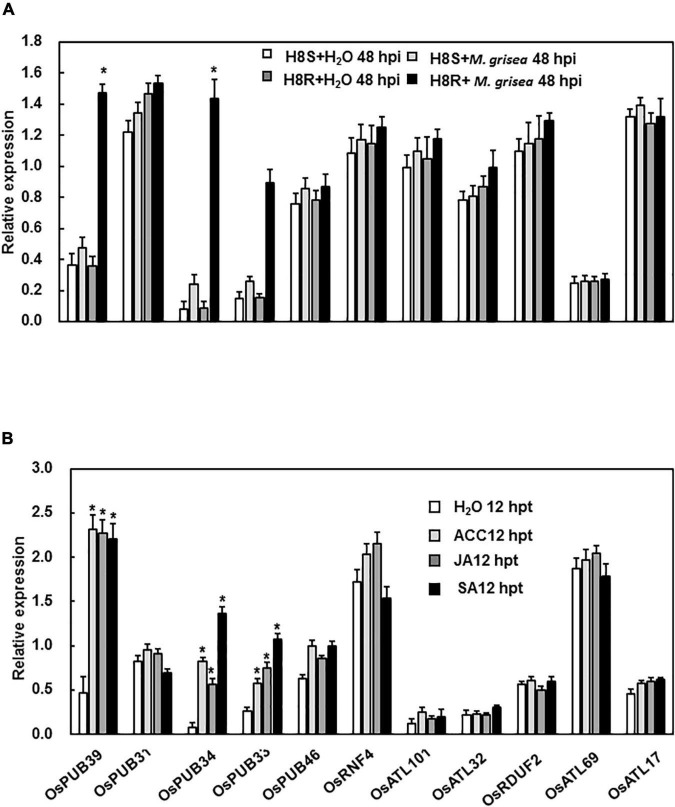
Expression patterns of E3 ubiquitin ligase genes in response to infection by *M. grisea* and hormone treatment. **(A)** Expression of E3 ubiquitin ligase genes in response to *M. grisea*. Leaves of H8R and H8S seedlings were sprayed with a solution of *M. grisea* spores. **(B)** Expression of E3 ubiquitin ligase genes in response to signaling hormones. Leaves of 4-week-old Yuanfengzao seedlings were treated with 1.5 mM SA, 100 μM JA, and 100 μM ACC solutions by spraying. JA and ACC solutions were prepared in 0.1% ethanol, and SA solution was prepared in water. The same volume of 0.1% ethanol or sterile distilled water was used as control. The samples were harvested at specific time points for the analysis of gene expression. Expression levels were presented as multiples of the *OsActin* expression level, which was used as standard. Data presented are the means ± SD from three independent experiments, and * above the columns indicate significant differences between the treated plants and control at *p* < 0.05 level.

By conducting searches on BLAST-P of the rice genome database using the characterized 11 *Arabidopsis* genes as queries, the corresponding gene sequences were obtained (Supplementary Material). To test whether these 11 genes functioned in response to stress, the expression patterns of these genes were analyzed following inoculation with *M. grisea* and treatment with hormone molecules. The expression of *OsPUB39*, *OsPUB34*, and *OsPUB33* was strongly induced by the inoculation of *M. grisea* in incompatible interaction, whereas the expression of other genes showed no significant difference between the treatment and control at the time point we test (Figure 1A). Furthermore, the expression levels of *OsPUB39*, *OsPUB34*, and *OsPUB33* were strongly induced by the hormones SA, JA, and ACC, whereas the expression levels of other genes were unaffected (Figure 1B).

### The Expression Patterns of E3 Ubiquitin Ligase Genes in Response to Abiotic Stress and Abscisic Acid

**FIGURE 2:**
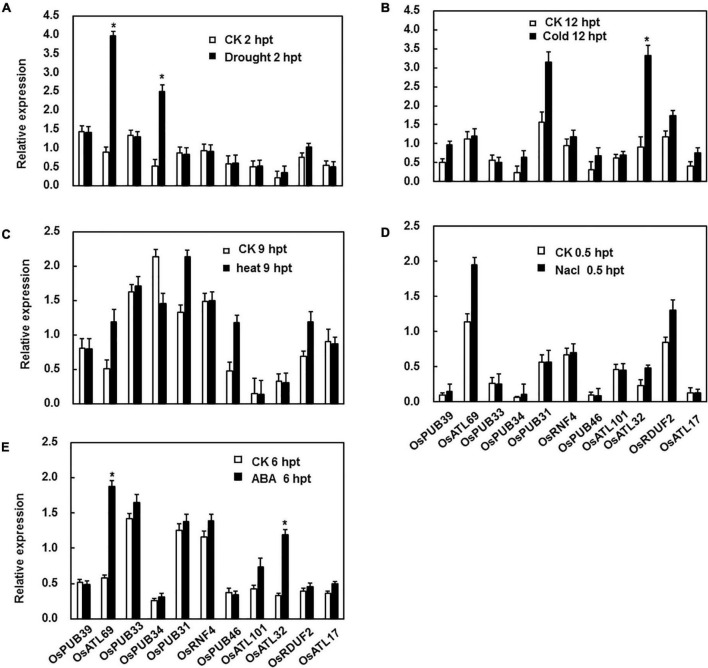
Expression patterns of E3 ubiquitin ligase genes in response to abiotic stress and ABA treatment. **(A)** For drought stress, the hydroponic 3-week-old plants were placed on the frame floor in the greenhouse after the water on the surface of the root was dried by filter paper. For cold **(B)** and heat stress **(C)**, 3-week-old plants were placed in a climatic cabinet at 4°C and 42°C. **(D)** For salt stress, hydroponic 3-week-old plants were placed in a 200 mM NaCl solution. **(E)** Expression patterns of E3 ubiquitin ligase genes in response to ABA. Leaves of 4-week-old Yuanfengzao seedlings were sprayed with 100 μM ABA solution. Ethanol (0.1%) was used as the control. The samples were harvested at specific time points for the analysis of gene expression. Expression levels were presented as multiples of the *OsActin* expression level, which was used as a standard. Data presented are the means ± SD from three independent experiments, and * above the columns indicate significant differences between the treated and control plants at *p* < 0.05 level.

As reported previously, PUB genes were involved in response to abiotic stresses, including drought, cold, heat, and salinity, to different degrees (Lu et al., 2020). Hence, drought, salinity, cold, and heat stresses were selected for the analysis of the expression patterns under abiotic stress. In the drought stress, the expression patterns of all genes except two (*OsATL69* and *OsPUB33*) showed no change. The expression of these two genes increased dramatically 2 h after treatment (Figure 2A). Under cold stress, the expression level of only *OsATL32* was strongly induced 12 h after treatment (Figure 2B). Under heat and salinity stresses, the expression levels of none of the genes were significantly affected (Figures 2C,D). ABA is a well-known stress-related hormone in plants and is involved in the responses to abiotic stress. We tested the expression patterns of E3 ubiquitin ligase genes following ABA treatment and observed that expression levels of OsATL69 and OsATL32 were significantly affected by ABA treatment at the time point we test (Figure 2E).

### BMV:OsPUB39-Infiltrated Plants Showed Decreased Resistance to *M. grisea* When Compared With BMV:00-Infiltrated Plants, Whereas BMV:OsPUB34- and BMV:OsPUB33-Infiltrated Plants Showed Increased Resistance

**FIGURE 3:**
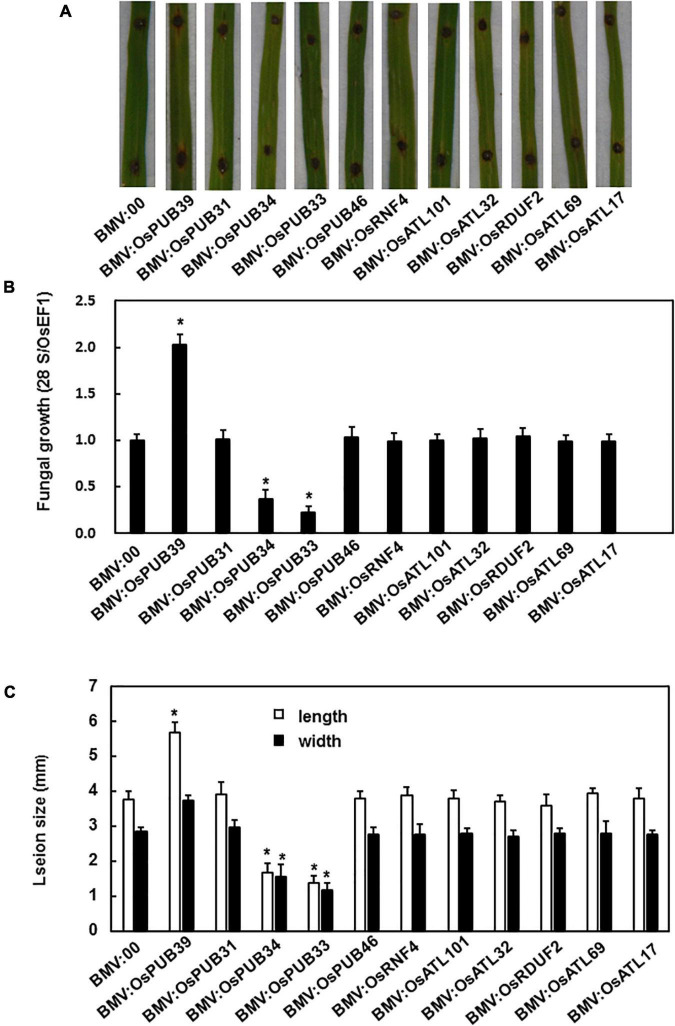
BMV:OsPUB34- and BMV:OsPUB33-infiltrated seedlings showed significantly increased resistance to *M. grisea* when compared with the control, whereas BMV:OsPUB39-infiltrated seedlings showed decreased resistance to *M. oryzae*. **(A)** The lesions on the rice leaves 7 days after inoculation. **(B)** The quantities of bacteria in leaves of BMV:target gene- and BMV:00-infiltrated seedlings. **(C)** The length and width of the lesions on BMV:target gene- and BMV:00-infiltrated seedlings. *Above the columns indicate significant differences between BMV:target gene- and BMV:00-infiltrated seedlings at *p* < 0.05 level.

We explored the possible role of the tested genes in the resistance to *M. grisea* by comparing the phenotype of BMV:target gene-infiltrated and BMV:00-infiltrated plants after the inoculation of *M. grisea*. The efficiency of silencing was tested before inoculation, and the most efficiently silencing plants were selected for the disease assay (Supplementary Material). After 7 days, the BMV:OsPUB39-infiltrated plants showed a more severe disease phenotype with larger lesion size and more fungi growth when compared with control, whereas the BMV:OsPUB34- and BMV:OsPUB33-infiltrated plants showed lighter disease phenotype with smaller lesion size and less fungal growth (Figure 3).

**FIGURE 4:**
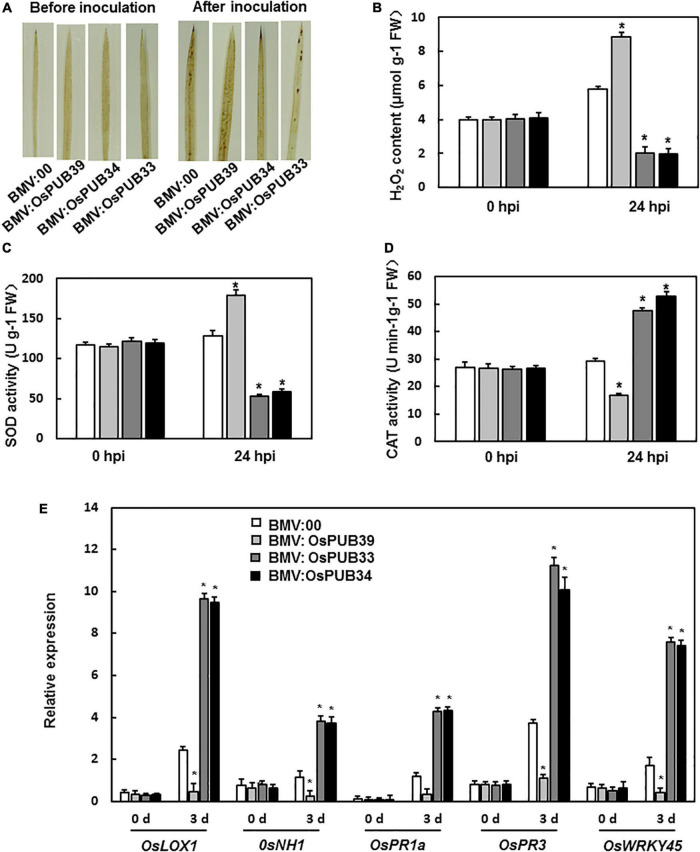
ROS accumulation and the expression levels of defense-related genes in BMV:target gene- and BMV:00-infiltrated seedlings before and after *M. oryzae* inoculation. **(A)** DAB staining of leaves from BMV:target gene- and BMV:00-infiltrated seedlings before and after *M. oryzae* inoculation. **(B)** H_2_O_2_ content in BMV:target gene- and BMV:00-infiltrated seedlings before and after *M. oryzae* inoculation. SOD activity **(C)** and CAT activity **(D)** in BMV:target gene- and BMV:00-infiltrated seedlings before and after *M. oryzae* inoculation. **(E)** Expression levels of defense-related genes were shown as multiples of the *OsActin* expression level, which was used as a standard. Data presented are the means ± SD from three independent experiments, and * above the columns indicate significant differences between BMV:target gene- and BMV:00-infiltrated seedlings at *p* < 0.05 level.

To investigate the mechanism of function of *OsPUB39*, *OsPUB34*, and *OsPUB33*’s in the resistance to *M. grisea*, we analyzed the ROS accumulation and the expression levels of defense-related genes. There was no significant difference in ROS accumulation between BMV: OsPUB39-, BMV: OsPUB34-, BMV: OsPUB33-, and BMV:00-infiltrated seedlings before *M. grisea* inoculation. However, after inoculation with *M. grisea*, the BMV:OsPUB34- and BMV:OsPUB33-infiltrated seedlings accumulated less ROS than the BMV:00-infiltrated seedlings, whereas BMV:OsPUB39-infiltrated seedlings accumulated more ROS (Figure 4A). Similar results were observed in terms of H_2_O_2_ content. After *M. grisea* inoculation, H_2_O_2_ content in BMV:OsPUB34- and BMV:OsPUB33-infiltrated seedlings was lower than that in BMV:00-infiltrated seedlings and higher in BMV:OsPUB39-infiltrated seedlings (Figure 4B). The activities of SOD and CAT were analyzed to investigate the reason behind the changed H_2_O_2_ content in BMV: OsPUB39-, BMV: OsPUB34-, and BMV:OsPUB33-infiltrated seedlings. Before *M. grisea* inoculation, SOD activity and CAT activity in the BMV:target gene- and BMV:00-infiltrated seedlings were not significantly different (Figures 4C,D). After *M. grisea* inoculation, SOD activity in BMV:OsPUB34- and BMV:OsPUB33-infiltrated seedlings decreased, whereas CAT activity increased when compared with BMV:00-infiltrated seedlings. In contrast, SOD activity in BMV:OsPUB39-infiltrated seedlings increased, whereas CAT activity decreased when compared with BMV:00-infiltrated seedlings after *M. grisea* inoculation (Figures 4C,D).

We also analyzed the expression levels of defense-related genes. After *M. grisea* inoculation, the expression levels of *OsLOX1*, *OsPR3*, *OsNH1*, *OsPR1a*, and *OsWRKY45* decreased in BMV:*OsPUB39*-infiltrated plants and increased in BMV:OsPUB34- and BMV:OsPUB33-infiltrated seedlings when compared to the control (Figure 4E). These results indicated that *OsPUB39*, *OsPUB34*, and *OsPUB33* were involved in the resistance to *M. grisea*, possibly through the regulation of the accumulation of ROS and expression of defense-related genes.

### The BMV:OsPUB33-Infiltrated Plants Showed Increased Tolerance to Drought Stress, Whereas the BMV:OsATL69-Infiltrated Plants Showed Decreased Tolerance

**FIGURE 5:**
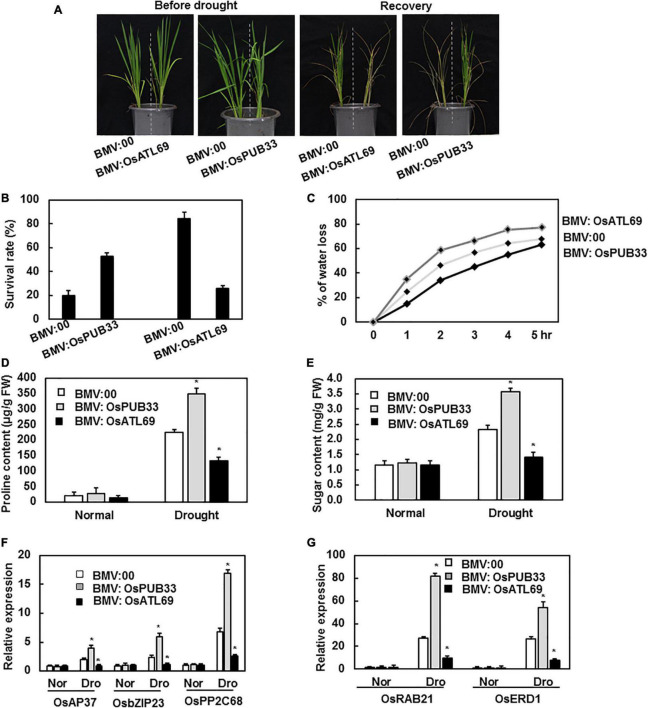
BMV:OsATL69-infiltrated seedlings showed decreased tolerance to drought stress while BMV:OsPUB33-infiltrated seedlings showed increased tolerance to drought stress. The BMV:OsATL69- and BMV:00-infiltrated seedlings in the same pot were withheld watering for 10 days and recovered with normal watering for another 12 days. The phenotype **(A)** and the survival rate **(B)** of BMV:target gene- and BMV:00-infiltrated seedlings after suffered drought stress. BMV:OsATL69- and BMV:00-infiltrated seedlings in the same pot were withheld watering for 10 days and recovered with normal watering for another 12 days. And BMV:OsPUB33- and BMV:00-infiltrated seedlings in the same pot which were withheld watering for 15 days and recovered with normal watering for another 12 days. **(C)** Water loss of BMV:OsATL69- and BMV:00-infiltrated seedlings after exposure to drought stress for different times. Proline content **(D)** and sugar content **(E)** of BMV: OsATL69-, BMV: OsPUB33-, and BMV:00-infiltrated seedlings after exposure to drought stress for 10 days. **(F,G)** The expression levels of drought-responsive genes were upregulated in BMV:OsPUB33-infiltrated plants and downregulated in BMV:OsATL69- infiltrated plants, when compared to the control after drought stress. Expression levels were shown as multiples of the *OsActin* expression level, which was used as a standard. Data presented are the means ± SD from three independent experiments, and * above the columns indicate significant differences between BMV:target genes- and BMV:00-infiltrated plants at *p* < 0.05 level.

To explore the possible function of the 11 genes in response to abiotic stress, we compared the phenotype of BMV:target gene- and BMV:00-infiltrated plants after exposure to abiotic stress. None of the plants showed a significant difference in tolerance to drought stress, except BMV:OsATL69- and BMV:OsPUB33-infiltrated plants. The BMV:OsPUB33-infiltrated plants showed increased tolerance to drought stress, whereas BMV:*OsATL69*-infiltrated plants showed decreased tolerance when compared with control (Figure 5A). The survival rate, water loss, proline content, and sugar content decreased significantly in BMV:OsATL69-infiltrated plants and increased significantly in BMV:OsPUB33-infiltrated plants (Figures 5B–E). We also tested the expression levels of drought-responsive genes and observed that their expression decreased in BMV:OsATL69-infiltrated plants and increased in BMV:OsPUB33- infiltrated plants (Figures 5F,G).

### BMV:OsATL32-Infiltrated Plants Decreased the Tolerance to Cold Stress

**FIGURE 6:**
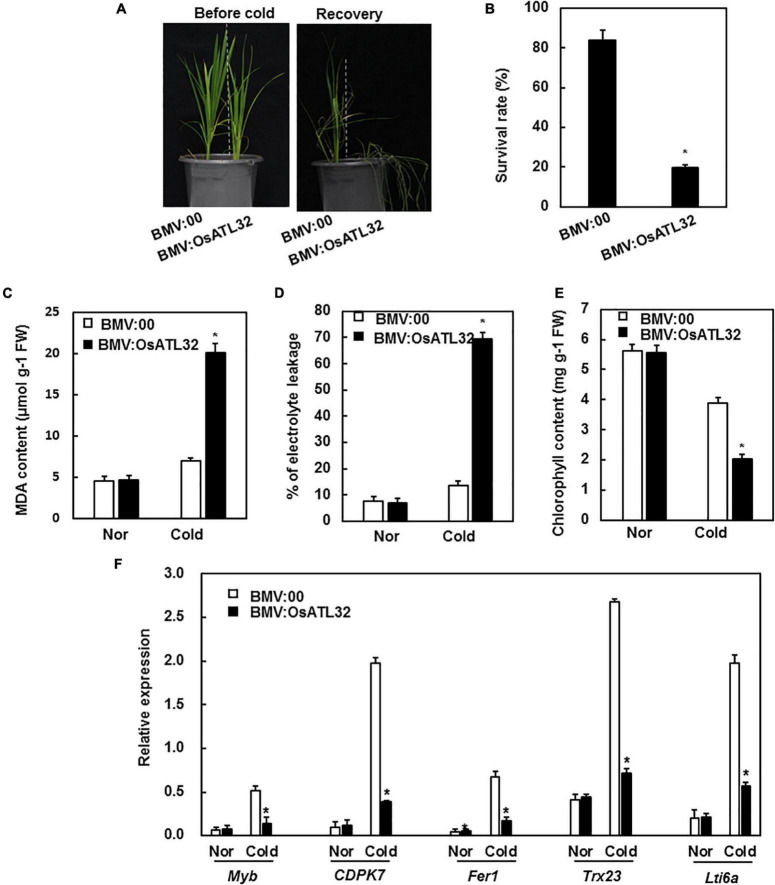
BMV:OsATL32-infiltrated seedlings showed decreased tolerance to cold stress when compared to the control. Four-week-old BMV:OsATL32- and BMV:00-infiltrated seedlings in the same pot were placed in a growth chamber at 4°C for 2 days, then transferred to normal growth conditions. Seven days later, the survival rate, MDA content, chlorophyll content, electrolyte leakage, and expression levels of cold-responsive genes were analyzed. **(A)** The phenotype of BMV: OsATL32-infiltrated plants after exposure to cold stress. **(B)** The survival rates were recorded. MDA content **(C)**, electrolyte leakage **(D)**, and chlorophyll content **(E)** were analyzed in BMV:OsATL32- and BMV:00-infiltrated seedlings with or without exposure to cold stress. **(F)** Expression of cold-responsive genes in BMV:OsATL32- and BMV:00-infiltrated seedlings with or without exposure to cold stress. Expression levels of cold-responsive genes were analyzed using qRT-PCR with gene-specific primers. Data presented are the means ± SD from three independent experiments, and * above the columns indicate significant differences between BMV:OsATL32- and BMV:00- infiltrated plants at *p* < 0.05 level.

Under cold stress, BMV:OsATL32-infiltrated plants showed decreased resistance when compared to the control (Figure 6A). The survival rate of BMV:OsATL32- infiltrated plants was 19.62 and 83.65% in control (Figure 6B). The MDA content and electrolyte leakage in BMV:OsATL32-infiltrated plants increased when compared to control (Figures 6C,D), whereas the chlorophyll content in BMV:OsATL32- infiltrated plants decreased (Figure 6E). Next, the expression levels of cold-responsive genes were analyzed and were observed to decrease significantly in BMV:OsATL32-infiltrated plants when compared to the control (Figure 6F).

## Discussion

E3 ubiquitin ligases play an important role in the ubiquitin-proteasome pathway, which is among the most important protein degradation pathway in eukaryotic organisms (Pickart and Eddins, 2004; Wang and Deng, 2011). There are several E3 ubiquitin ligases in plants, for example, at least 60 in *Arabidopsis*. The study of E3 ubiquitin ligases is a topic of immense interest, and the functions of E3 ubiquitin ligases are explored regularly. However, the functions of E3 ubiquitin ligases in rice were rarely studied. In this research, 11 E3 ubiquitin ligase genes from *Arabidopsis*, which were hypothesized to function in response to biotic or abiotic stresses, were selected, and the homologous genes in rice were found.

Plants are unavoidably exposed to abiotic and biotic stresses and have formed sophisticated mechanisms to adapt to such adverse conditions. Phytohormones play an important role in helping plants to adapt to environmental situations (Verma et al., 2016). Several phytohormone signaling pathways are dependent on the ubiquitin-proteasome system, specifically E3 ubiquitin ligases that can perceive and initiate signal transduction (Kelley, 2018; Tal et al., 2020). Therefore, the expression patterns of these 11 genes following treatment with hormones such as JA, ACC, and SA were analyzed first. The expression levels of only three genes, OsPUB39, OsPUB34, and OsPUB33, were induced by treatment with JA, ACC, and SA (Figure 1B). This result indicates that these genes may function in response to biotic stress. Next, the functions of these genes in response to *M. grisea* were analyzed. The expression levels of OsPUB39, OsPUB34, and OsPUB33 were induced by *M. grisea*, and the silencing of these three genes altered the resistance to *M. grisea* (Figures 1A, 3). OsPUB34 and OsPUB33 negatively regulated the resistance to *M. grisea*. The functions of OsPUB34 and OsPUB33 in rice in response to biotic stress were similar to that of AtPUB22 and AtPUB23 in *Arabidopsis*, with both sets of genes being negative regulators of biotic stresses (Cho et al., 2008; Trujillo et al., 2008). However, OsPUB31 appeared not to affect the resistance to *M. grisea*. CMPG1, a highly related gene to *Arabidopsis* PUB20 and PUB21, positively regulated the response to disease resistance in tomato and tobacco (Gonzalez et al., 2006). The OsPUB39 probably positively regulated the resistance to *M. grisea* (Figure 3). These results further confirmed previous reports that E3 ubiquitin ligases regulated the resistance to biotic stress, either positively or negatively (Ni et al., 2010; He et al., 2015; Wang et al., 2016; You et al., 2016; Chen et al., 2017; Kim et al., 2021).

ROS production is important for the activation of immune responses against pathogen infection, and the expression levels of defense-related genes are closely related to disease resistance (Lehmann et al., 2015; Qi et al., 2018; Segal and Wilson, 2018; Waszczak et al., 2018). E3 ubiquitin ligase also regulated the resistance to biotic stress in these two ways (Yaeno and Iba, 2008; Zhou and Zeng, 2018). Silencing of APIP6 resulted in reduced resistance to *M. oryzae* in rice by reducing the generation of flg22-induced ROS and suppressing the expression of defense-related genes (Park et al., 2012). In our study, ROS accumulation and the expression levels of defense-related genes were analyzed to explore the mechanism for the altered resistance by gene silencing. The silencing of OsPUB39 resulted in increased ROS accumulation and H_2_O_2_ content, while the silencing of OsPUB34 and OsPUB33 resulted in less ROS accumulation and H_2_O_2_ content (Figures 4A,B). Plants have a highly efficient system for maintaining ROS homeostasis (Mittler et al., 2004). They possess two mechanisms of scavenging ROS, one is by small molecules (including glutathione, ascorbic acid, flavonoids, alkaloids, and carotenoids), and the other is by detoxifying enzymes, including superoxide dismutase (SOD), catalase (CAT), peroxidase, and peroxiredoxins (Lehmann et al., 2009). The activities of SOD and CAT were analyzed to explain the altered ROS accumulation. The altered SOD and CAT activities in BMV:target gene-infiltrated seedlings may explain the change in ROS accumulation (Figures 4C,D). The expression levels of defense-related genes were also analyzed. *LeATL6* regulated elicitor-activated defense responses via a JA-dependent signaling pathway in tomato (Hondo et al., 2007). ASK1/ASK2 and cullin-1 formed the SCF-ubiquitin ligase complex, as the signaling receptor of JA, through the degradation of the inhibitor JAZ of the JA pathway, to activate the expression of JA-response genes (Devoto et al., 2002; Xu et al., 2002; Ren et al., 2005; Chini et al., 2007; Thines et al., 2007). MdPUB29 increased the resistance to *Botryosphaeria dothidea* by the SA pathway (Han et al., 2019). Therefore, SA-responsive genes, JA-responsive genes, and *OsWRKY45* (a positive regulator in response to fungal pathogens) were selected. The expression level of *OsWRKY45* decreased in BMV:OsPUB39-infiltrated plants and increased in the BMV:OsPUB34- and BMV:OsPUB33-infiltrated plants. The expression levels of JA-responsive genes (*OsLOX1* and *OsPR3*) and SA-responsive genes (*OsNH1* and *OsPR1a*) were decreased in BMV:OsPUB39-infiltrated plants when compared to control and increased in BMV:OsPUB34- and BMV:OsPUB33-infiltrated plants (Figure 4E). These results indicate that OsPUB39, OsPUB34, and OsPUB33 were involved in response to *M. grisea*, possibly through SA and JA/ET pathways.

Drought and cold are major abiotic stresses that severely affect plant growth and productivity. E3 ubiquitin ligases have been reported to be positively or negatively involved in response to drought stress. OsiSAP7 negatively regulated ABA-stress signaling and imparted sensitivity to drought stress in *Arabidopsis* (Sharma et al., 2015). AIRP1 positively regulated the response to drought. Its overexpression in plants increased stomatal closure, ROS accumulation, expression of drought-responsive genes, and ABA-responsive bZIP transcript factor (Ryu et al., 2010). *SDIR1* positively regulated the ABA pathway. The knockout mutants were less sensitive to ABA, whereas the overexpression plants showed increased stomatal closure and resistance to drought in *Arabidopsis* (Zhang et al., 2007). Similarly, the plants with overexpression of *RHA2a/RHA2b* were highly sensitive to ABA, with increased stomatal closure, decreased water loss, and increased resistance to drought (Bu et al., 2009; Li et al., 2011). Wheat TaPUB1 positively regulated the tolerance to drought stress by improving antioxidant capability (Zhang et al., 2017). Our study showed that the expression levels of *OsATL69* and *OsPUB33* were induced by drought stress (Figure 2A). OsATL69 positively regulated the tolerance to drought stress (Figure 5), which was in line with the previous reports that AtATL78 acted as positive regulators of drought stress (Cho et al., 2008; Trujillo et al., 2008). On the other hand, OsPUB33 negatively regulated the tolerance to drought stress (Figure 5), consistent with previous reports that AtPUB22, AtPUB23, AtPUB24, and OsPUB41 acted as negative regulators of drought stress (Cho et al., 2008; Trujillo et al., 2008; Seo et al., 2021). To explore the reason for the altered tolerance to drought stress due to the silencing of *OsATL69* or *OsPUB33*, the proline content, sugar content, and expression levels of drought-responsive genes were analyzed. Proline content and sugar content in BMV:OsATL69-infiltrated plants decreased under drought stress when compared to the control but increased in BMV:OsPUB33-infiltrated plants (Figures 5D,E). The expression levels of drought-responsive genes increased in BMV:OsPUB33-infiltrated plants and decreased in BMV:OsATL69- infiltrated plants compared to the control (Figures 5F,G). These results indicated that *OsATL69* and *OsPUB33* conferred tolerance to drought stress, possibly through the regulation of proline content, sugar content, and the expression levels of drought-responsive genes.

E3 ubiquitin ligases are also involved in the tolerance to cold stress. AtATL78 and AtATL80 negatively regulated the tolerance to cold stress in *Arabidopsis* (Lee et al., 2001; Kim and Kim, 2013; Suh and Kim, 2015). OsDIRP1 positively regulated the tolerance to cold stress in rice (Cui et al., 2018). We observed that the expression of *OsATL32* was induced by cold stress (Figure 2B). The BMV:OsATL32-infiltrated seedlings showed decreased resistance to cold resistance when compared to control (Figure 6A), with a lower survival rate and chlorophyll content (Figures 6B,E), but higher MDA content and electrolyte leakage (Figures 6C,D). Moreover, the expression of cold-responsive genes in BMV:OsATL32-infiltrated seedlings was downregulated when compared to the control (Figure 6E). These results indicate that OsATL32 regulated the tolerance to cold stress, possibly through MDA content and the expression levels of cold-responsive genes.

ABA is a critical signaling mediator that regulates diverse biological processes in various organisms (Kumar et al., 2019). Plants regulate response to abiotic stress via two pathways, one is ABA-dependent, and the other is ABA-independent. E3 ubiquitin ligases regulate the tolerance to abiotic stresses dependent or independent of ABA. AtARRE negatively regulates ABA signaling in *Arabidopsis thaliana* (Wang et al., 2018). *PeCHYR1* elevates the tolerance to drought stress by ABA-induced stomatal closure via ROS production in *Populus euphratica* (He et al., 2018). Rma1H1 responds to drought by mediating the ubiquitination of water channel protein isoenzyme PIP2;1 to downregulate the expression of the water channel protein, independent of ABA (Lee et al., 2009; Son et al., 2009; Bae et al., 2011). Our results indicate that the expression levels of *OsATL69* and *OsATL32* were induced by ABA, whereas those of *OsPUB33* were not (Figure 2E). This may indicate that the regulation of response by *OsATL69* and *OsATL32* to abiotic stress was ABA-dependent, whereas that of *OsPUB33* was ABA-independent. This need further experiment by ABA content or ABA treatment on infiltrated seedlings.

E3 ubiquitin ligases have been reported to function in response to heat stress. AtSAP5 conferred tolerance to heat stress (Kim et al., 2015). *AtPPRT1* increased the tolerance to heat stress in *Arabidopsis* (Liu et al., 2020). SlSIZ1 positively regulated the tolerance to heat stress in tomato (Zhang et al., 2018), whereas HTD1 negatively regulated thermotolerance in *Arabidopsis* (Kim et al., 2014). However, in our study, the expression levels of none of the E3 ubiquitin ligase genes were observed to be induced by heat stress (Figure 2C). Also, because of the elimination of VIGS by high temperature, we could not research its function in response to heat stress. The possible roles of these 11 E3 ubiquitin ligase genes in the resistance to heat stress can be explored using transgenic lines.

## Data Availability Statement

The original contributions presented in the study are included in the article/Supplementary Materials, further inquiries can be directed to the corresponding author/s.

## Author Contributions

FS conceived the study. HZ and MJ designed the experiments. HZ and DZ performed the experiments. HZ and FS analyzed the data. MJ drafted the manuscript. All authors read and approved the final manuscript.

## Conflict of Interest

The authors declare that the research was conducted in the absence of any commercial or financial relationships that could be construed as a potential conflict of interest.

## Publisher’s Note

All claims expressed in this article are solely those of the authors and do not necessarily represent those of their affiliated organizations, or those of the publisher, the editors and the reviewers. Any product that may be evaluated in this article, or claim that may be made by its manufacturer, is not guaranteed or endorsed by the publisher.
