# Supplementary material for: Expression Patterns and Functional Analysis of 11 E3 Ubiquitin Ligase Genes in Rice
Source: Front Plant Sci. 2022 Mar 2;13:840360. doi: 10.3389/fpls.2022.840360 (PMC8924586; doi:10.3389/fpls.2022.840360)
Supplement: Supplementary file 1 [file Data_Sheet_1.PDF]

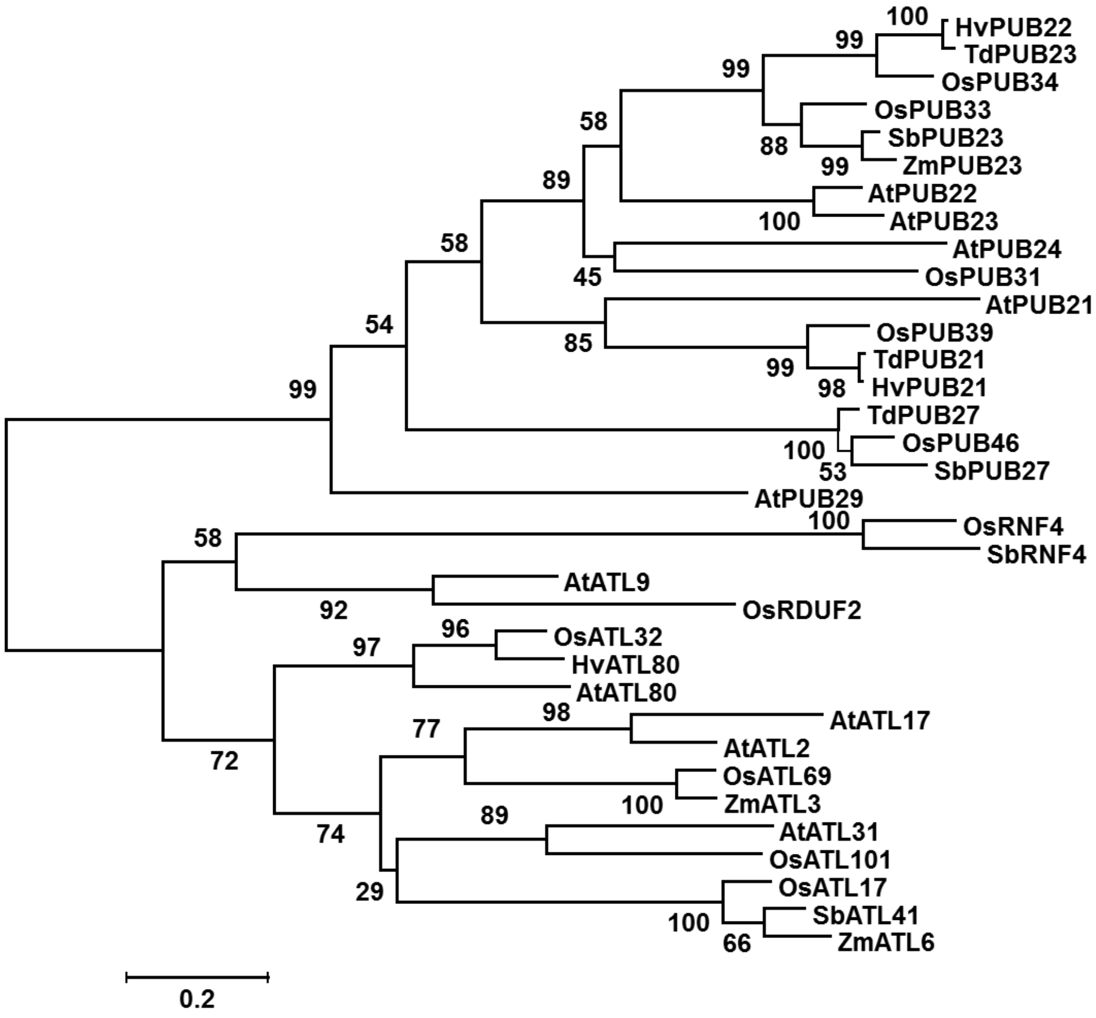

FIGURE 1 | Phylogenetic tree of 11 E3 ubiquitin ligase genes studied in this research. The phylogenetic tree was drawn by the neighbor-joining method.

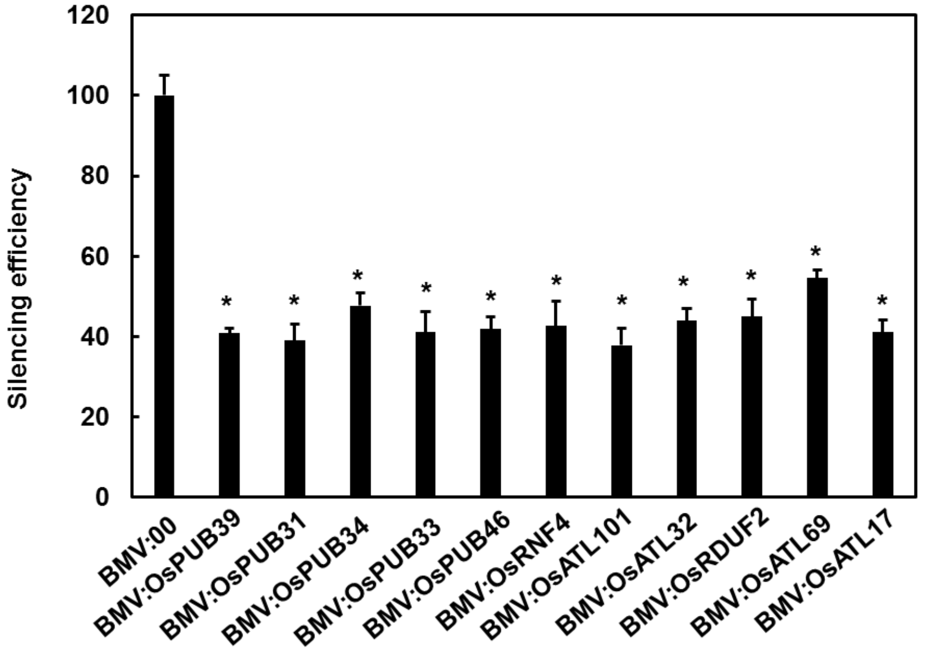

FIGURE 2 | The silencing efficiency of the BMV:target gene-infiltrated plants.
